# Predicting isocitrate dehydrogenase mutation status in acute myeloid leukemia from gene expression profiles by machine learning

**DOI:** 10.1093/nargab/lqag058

**Published:** 2026-06-08

**Authors:** Ina Jung, Anne-Laure Vitte, Florent Chuffart, Simon Chevalier, Pascal Mossuz, Saadi Khochbin, Ekaterina Bourova-Flin

**Affiliations:** Université Grenoble Alpes, INSERM U1209, CNRS UMR 5309, Institute for Advanced Biosciences, 38000, Grenoble, France; Université Grenoble Alpes, INSERM U1209, CNRS UMR 5309, Institute for Advanced Biosciences, 38000, Grenoble, France; Université Grenoble Alpes, INSERM U1209, CNRS UMR 5309, Institute for Advanced Biosciences, 38000, Grenoble, France; Université Grenoble Alpes, INSERM U1209, CNRS UMR 5309, Institute for Advanced Biosciences, 38000, Grenoble, France; Université Grenoble Alpes, CHU Grenoble Alpes, Department of Biological Hematology, 38000, Grenoble, France; Université Grenoble Alpes, INSERM U1209, CNRS UMR 5309, Institute for Advanced Biosciences, 38000, Grenoble, France; Université Grenoble Alpes, CHU Grenoble Alpes, Department of Biological Hematology, 38000, Grenoble, France; Université Grenoble Alpes, INSERM U1209, CNRS UMR 5309, Institute for Advanced Biosciences, 38000, Grenoble, France; Université Grenoble Alpes, INSERM U1209, CNRS UMR 5309, Institute for Advanced Biosciences, 38000, Grenoble, France

## Abstract

We developed machine-learning models to predict isocitrate dehydrogenase (IDH) mutation status in acute myeloid leukemia (AML) from gene expression profiles and to reconstruct missing IDH annotations across public datasets. Transcriptomic data from 19 cohorts (5844 samples) were harmonized using batch correction, and 1546 samples with known IDH status were used to train a feed-forward neural network and a logistic regression (LR) classifier within a nested cross-validation framework, followed by independent validation in the TCGA-LAML dataset. The LR model showed superior performance, achieving receiver operating characteristic area under the curve $=$ 0.994 $\pm$ 0.007, accuracy $=$ 0.983 $\pm$ 0.006, balanced accuracy $=$ 0.979 $\pm$ 0.005, sensitivity for the IDH-mutant (IDH-MUT) class $=$ 0.972 $\pm$ 0.010, and specificity $=$ 0.986 $\pm$ 0.008, and correctly classified all IDH-MUT cases in the independent cohort. Applying the final model to samples lacking annotations enabled reconstruction of IDH status for 4148 AML cases, expanding the number of molecularly characterized transcriptomes available for downstream analyses. Predicted groups recapitulated known IDH-associated transcriptional signatures, supporting biological validity. This work demonstrates that IDH mutation status can be accurately inferred from transcriptomic data alone and provides a scalable framework to recover missing genomic annotations, thereby enhancing the utility of public AML resources for large-scale biological and translational research.

## Graphical Abstract

**Graphical Abstract:**
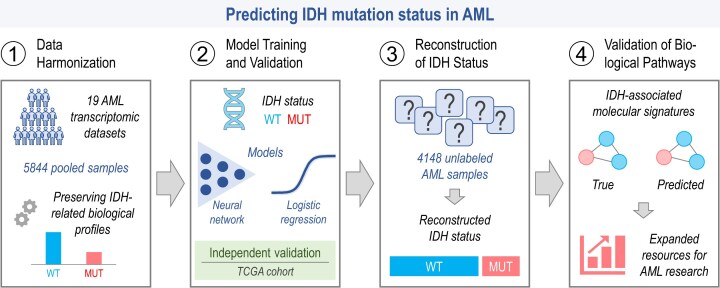

## Introduction

Isocitrate dehydrogenase (IDH) mutations represent recurrent oncogenic events across multiple cancer types, including acute myeloid leukemia (AML) and glioma [1]. IDH enzymes are key metabolic regulators involved in cellular redox balance and the tricarboxylic acid (TCA) cycle. Under physiological conditions, wildtype (WT) IDH1 (localized in the cytoplasm and peroxisomes) and IDH2 (localized in the mitochondrial matrix) catalyze the NADP$^+$-dependent oxidative decarboxylation of isocitrate to $\alpha$-ketoglutarate ($\alpha$-KG).

Cancer-associated mutations in IDH1 and IDH2 confer a neomorphic enzymatic activity that drives the reduction of $\alpha$-KG to the oncometabolite D-2-hydroxyglutarate (2-HG). Consequently, IDH1 mutations predominantly lead to cytoplasmic accumulation of 2-HG, whereas IDH2 mutations initially generate 2-HG within mitochondria, from which it can subsequently be exported to the cytosol [2, 3]. Regardless of its subcellular origin, elevated intracellular 2-HG acts as a competitive inhibitor of $\alpha$-KG-dependent dioxygenases, including TET family DNA hydroxylases and Jumonji-C domain histone demethylases. Inhibition of these enzymes results in widespread alterations in cellular metabolism, DNA and histone methylation, and chromatin organization [4]. Through these mechanisms, IDH mutations profoundly reshape the epigenetic landscape, promote aberrant self-renewal programs, and contribute to impaired differentiation and malignant transformation.

AML is an aggressive hematologic malignancy characterized by clonal expansion and accumulation of immature myeloid progenitor cells (blasts) in the bone marrow, leading to disruption of normal hematopoiesis [5]. AML accounts for ∼30% of adult leukemias and primarily affects older individuals, with a median age at diagnosis of about 65–70 years [6]. Although AML is the most common acute leukemia in adults, it remains relatively rare among all malignancies, representing ∼1% of all cancer diagnoses worldwide, with an incidence of about four cases per 100 000 individuals per year, accordingly to the American Cancer Society.

Large cohort studies indicate that IDH1 or IDH2 mutations occur in roughly 15%–20% of adult AML cases, including ∼6%–16% for IDH1 and 8%–19% for IDH2, with variability depending on cohort composition, age distribution, and cytogenetic risk group [7, 8]. IDH mutations define a distinct molecular AML subtype characterized by specific transcriptional, metabolic, and epigenetic features. These include enrichment of stem and progenitor transcriptional programs, frequent association with normal cytogenetics and co-mutations such as NPM1, DNMT3A, or SRSF2, and altered mitochondrial and lipid metabolism [9, 10]. Importantly, IDH-mutant (IDH-MUT) AML has become a therapeutically actionable entity with the development of selective IDH1 and IDH2 inhibitors, used as monotherapy or in combination with standard regimens [11, 12]. Furthermore, IDH-MUT AML cells undergo extensive metabolic rewiring, creating distinct metabolic dependencies that may be therapeutically exploitable [13, 14]. Accurate determination of IDH mutation status is therefore essential for biological characterization and clinical stratification of AML.

Despite its importance, IDH mutation status is frequently missing in publicly available AML transcriptomic datasets. Many legacy gene expression studies predate routine mutational profiling, while others provide incomplete or heterogeneous molecular annotations. Even when available, the relatively low prevalence of IDH mutations often results in small sample sizes, limiting statistical power and complicating integrative analyses. This scarcity constrains large-scale investigations of IDH-associated transcriptional programs and reduces the utility of public datasets for studying metabolic-epigenetic dysregulation in AML.

Previous studies have described transcriptional and epigenetic signatures associated with IDH mutations, including characteristic DNA hypermethylation patterns and dysregulation of differentiation-related genes [15–18]. However, these signatures were typically derived from limited cohorts and were not designed for systematic inference of IDH mutation status across heterogeneous datasets. Consequently, a scalable and robust method to infer IDH status directly from gene expression data remains an unmet need.

To address this gap, we developed two complementary machine-learning models—a linear logistic regression (LR) classifier and a nonlinear feed-forward neural network (NN)—to predict global IDH mutation status (mutant versus WT) from AML transcriptomic profiles. We then applied these models to multiple public AML datasets lacking IDH annotations, enabling systematic reconstruction of IDH status across cohorts. By resolving missing molecular annotations, this framework facilitates large-scale reuse of transcriptomic resources and provides a foundation for deeper investigation of IDH-driven metabolic and epigenetic programs in AML.

## Materials and methods

### Public datasets of AML

We analyzed 19 publicly available AML transcriptomic datasets obtained from the following repositories: the GDC portal (https://portal.gdc.cancer.gov/), NCBI GEO (https://www.ncbi.nlm.nih.gov/geo/), and cBioPortal for Cancer Genomics (https://www.cbioportal.org/). Detailed characteristics of all datasets are summarized in Table 1.

**Table 1.** Sample sizes of the AML datasets

Dataset	Technology	Source	Sample size	Samples with available IDH status
AML-OHSU-2022	RNA-seq	cBioPortal	654	117 IDH-MUT (19.3%),
				489 IDH-wildtype (IDH-WT) (80.7%)
BEATAML-1.0	RNA-seq	GDC portal	463	
GSE106291	RNA-seq	NCBI GEO	250	47 IDH-MUT (19.8%),
				190 IDH-WT (80.2%)
GSE111678	Microarrays	NCBI GEO	260	
GSE1159	Microarrays	NCBI GEO	285	
GSE13159	Microarrays	NCBI GEO	542	
GSE146173	RNA-seq	NCBI GEO	246	50 IDH-MUT (20.3%),
				196 IDH-WT (79.7%)
GSE165430	RNA-seq	NCBI GEO	268	
GSE17855	Microarrays	NCBI GEO	237	
GSE216738	RNA-seq	NCBI GEO	506	
GSE22845	Microarrays	NCBI GEO	154	
GSE232130	RNA-seq	NCBI GEO	362	
GSE253086	RNA-seq	NCBI GEO	136	
GSE297413	RNA-seq	NCBI GEO	264	
GSE37642	Microarrays	NCBI GEO	140	
GSE43176	Microarrays	NCBI GEO	104	
GSE61804	Microarrays	NCBI GEO	286	
GSE6891	Microarrays	NCBI GEO	536	71 IDH-MUT (15.5%),
				386 IDH-WT (84.5%)
TCGA-LAML	RNA-seq	GDC portal	151	29 IDH-MUT (19.3%),
				121 IDH-WT (80.7%)

Nine microarray datasets (GSE111678, GSE1159, GSE13159, GSE17855, GSE22845, GSE37642, GSE43176, GSE61804, GSE6891) were obtained with Affymetrix Human Genome Arrays U133 Plus 2.0 or U133A. Raw expression data were normalized using the Robust Multi-array Average method [19] followed by log$_2$ transformation.

For RNA-seq datasets, preprocessing depended on data availability. For GSE106291 and GSE146173, raw reads were aligned using STAR [20] and gene-level counts were obtained with HTSeq [21]. Expression values were converted to reads per kilobase of transcript per million reads mapped (RPKM) and log-transformed using log$_2$(1 + RPKM). For BEATAML-1.0, GSE165430, GSE216738, GSE232130, and TCGA-LAML, raw count data were downloaded and processed using the same normalization strategy. For AML-OHSU-2022, preprocessed RPKM values were used and log-transformed. For GSE253086 and GSE297413, transcripts per million (TPM) values were obtained and log-transformed using log$_2$(1 + TPM).

Among the ten RNA-seq datasets, raw counts were available for seven, whereas three datasets provided only preprocessed normalized values (RPKM or TPM), preventing uniform normalization across all datasets. For consistency, datasets with raw counts were normalized to RPKM, aligning with the AML-OHSU-2022 cohort, which was selected as reference due to its larger size and availability of IDH annotations.

### Identification of differently deregulated genes between the IDH-MUT and IDH-WT tumors

Differential analyses between the IDH-MUT versus IDH-WT tumor samples were performed in five AML datasets with available IDH mutation annotations, to identify significantly deregulated genes. For each gene and each dataset, expression levels between the tumor groups were compared using the Mann–Whitney U test. *P*-values were adjusted for multiple testing comparisons by the Benjamini–Hochberg procedure. Genes were considered significantly deregulated if they met the following criteria: false discovery rate (FDR) $< $ 0.05, and absolute fold change $> $ 2. Intersections of significantly upregulated and downregulated genes across datasets were then computed to identify robust IDH-associated transcriptional markers. Three genes, CD93, LGALS3BP, and ROGDI, were consistently downregulated in IDH-MUT samples in all datasets and were used as readout genes for subsequent batch-effect quality control.

### Preparation of a pooled AML dataset and batch-effect correction

Transcriptomes from all 19 datasets were combined into a single compendium for machine-learning analyses. To mitigate inter-study technical variability, batch effects were corrected using the pyComBat (v0.20) implementation [22]. The effectiveness of batch correction was assessed through multiple sensitivity analyses. Principal component analysis (PCA) was performed before and after correction using the full transcriptome. Expression distributions of the three readout genes (CD93, LGALS3BP, ROGDI) were examined pre- and post-correction. Additionally, differential expression analysis between IDH-MUT and IDH-WT samples was repeated in the pooled dataset to confirm preservation of biological signals. These analyses demonstrated that batch correction reduced technical variability while maintaining IDH-associated expression patterns.

Differences between normalization units (RPKM and TPM) may introduce systematic distributional shifts across datasets. Although pyComBat is not specifically designed to reconcile normalization-specific differences, it adjusts for nonbiological variability at the level of mean and variance. The impact of normalization heterogeneity was therefore evaluated through a dedicated sensitivity analysis (Supplementary Methods).

### Design and training of a NN predicting IDH status

To assess whether nonlinear models could capture additional predictive structure in transcriptomic data, we implemented a feed-forward NN (multilayer perceptron, MLP) to predict IDH mutation status from gene expression profiles. The model consisted of two hidden layers with ReLU activation functions and was trained using a binary cross-entropy loss with class weighting to account for imbalance between IDH-WT and IDH-MUT samples. Model development was performed within a nested cross-validation framework to ensure unbiased performance estimation. Gene expression features were standardized within each training fold, and early stopping was applied to prevent overfitting. A detailed description of the NN architecture, hyperparameter optimization, and training procedure is provided in the Supplementary Methods.

### Design and training of a LR model predicting IDH status

A linear LR classifier was used as a second predictive model, chosen for its interpretability, robustness in high-dimensional settings, and suitability for binary classification. Gene expression features were standardized using z-score normalization within each training fold to prevent information leakage. Model training and evaluation were implemented using the scikit-learn framework (Python).

#### Hyperparameter tuning and class imbalance handling

Model development was conducted using a nested stratified cross-validation strategy. The outer loop consisted of five stratified folds and was used to estimate generalization performance, while the inner loop of four stratified folds was used for hyperparameter optimization. Hyperparameters evaluated during grid search included the inverse regularization strength (from 1 to 10), the regularization parameters for pure L1 or pure L2 regularization, the maximum number of optimization iterations (100 or 500), and the minimum accepted gene expression variance threshold across training fold. Model selection within the inner loop was based on receiver operating characteristic (ROC) area under the curve (AUC), which served as the primary optimization criterion. To account for class imbalance between IDH-WT and IDH-MUT samples, class weights were automatically adjusted using a balanced weighting scheme.

#### Model performance evaluation and prediction

Model performance was evaluated on held-out test data using multiple complementary metrics, including ROC-AUC, accuracy, balanced accuracy, precision, recall and F1-score metrics, and Brier score. Results were summarized across outer cross-validation folds by calculating mean performance estimates. A final LR model was trained on the complete dataset using hyperparameters selected from the nested cross-validation procedure.

In the independent TCGA-LAML dataset, confidence intervals (CIs) for performance metrics were calculated as follows. Sensitivity and specificity were treated as binomial proportions and their 95% CIs were computed using the exact Clopper–Pearson method. For composite metrics (accuracy, balanced accuracy, and ROC-AUC), CIs were estimated using nonparametric bootstrap resampling with 1000 iterations.

### Gene set enrichment analysis

Gene set enrichment analysis (GSEA) was performed on the Hallmark (H), Curated (C2), and Gene Ontology (C5) collections of gene sets provided by the Broad Institute in the MSigDB database (https://www.gsea-msigdb.org/gsea), using the GSEA software GSEApy implemented in Python and available on the website [23].

## Results

### Design of a strategy for the establishment of models predicting IDH status

To develop predictive models of IDH mutation status, we implemented a multi-step analytical strategy. First, we generated a harmonized compendium by integrating transcriptomic data from 19 AML datasets, comprising 5844 samples. Batch effects were corrected using pyComBat, after which all samples were merged into a single dataset. To verify that the correction preserved biologically relevant signals, we identified genes consistently associated with IDH mutation status in the original uncorrected datasets (transcriptional readouts of IDH status) and confirmed that their expression patterns remained stable after batch adjustment.

In the second step, 1546 AML samples with known IDH status from four cohorts (AML-OHSU-2022, GSE106291, GSE146173, and GSE6891) were used to train and evaluate two classifiers—a feed-forward NN and a LR model—within a nested cross-validation framework. Model performance was assessed using several discrimination and calibration metrics across test folds. An independent set of 150 samples from the TCGA-LAML cohort with available IDH annotations was reserved for external validation of the selected model. This dataset was selected for independent validation because it enabled us to maximize the training sample size to optimize model performance while retaining a sufficiently large and independent cohort for reliable external validation.

In the third step, the final model was applied to the pooled AML dataset to infer IDH status for the 4148 samples lacking mutation annotations. Finally, we identified molecular signatures associated with IDH-MUT tumors in datasets with known labels and demonstrated that these signatures were consistently enriched or depleted in the corresponding predicted groups, supporting the biological validity of the classification.

### Three genes, CD93, LGALS3BP and ROGDI, represent stable transcriptional readouts associated with IDH mutation

**Figure 1.**
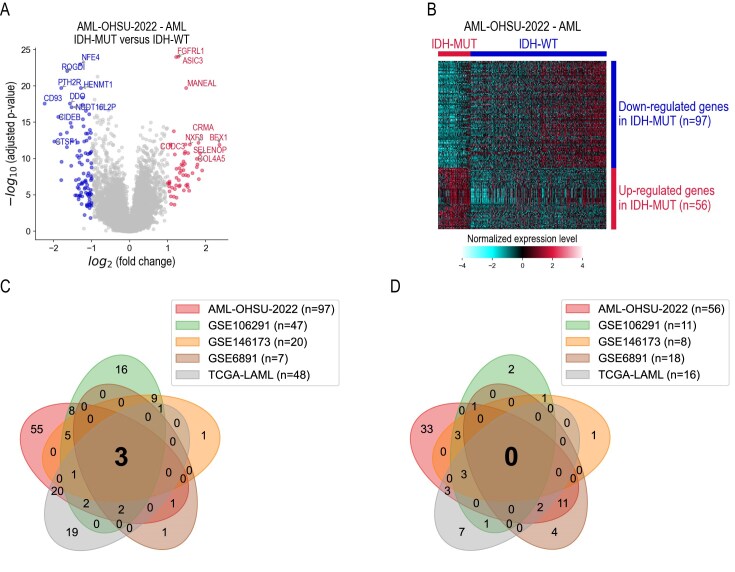
Transcriptomic differences between IDH-MUT and IDH-WT tumors across five AML cohorts. Differential gene expression analyses were performed between IDH-MUT and IDH-WT samples in five AML datasets with available mutation annotations. Differentially expressed genes were identified using the Mann–Whitney test with a FDR-adjusted *P*-value <.05 and an absolute fold change $> $ 2. (**A**) Volcano plot illustrating significantly upregulated and downregulated genes in IDH-MUT tumors from the AML-OHSU-2022 dataset. (**B**) Heatmap showing hierarchical clustering based on the differentially expressed genes in the AML-OHSU-2022 dataset. Samples were clustered using Euclidean distance with Ward’s linkage, and genes were clustered using Pearson correlation. (**C**) Intersection of genes significantly downregulated in IDH-MUT tumors across the five AML datasets. Three genes (CD93, LGALS3BP, and ROGDI) were consistently downregulated in all cohorts. (**D**) Intersection of genes significantly upregulated in IDH-MUT tumors across the five AML datasets. No genes were consistently upregulated in all cohorts.

To identify robust transcriptional markers of IDH mutations, we performed differential expression analyses between IDH-MUT versus IDH-WT samples in five AML datasets with available IDH mutation annotations (AML-OHSU-2022, GSE106291, GSE146173, GSE6891, and TCGA-LAML). For each gene and dataset, fold changes between group means, nominal *P*-values, and FDR-adjusted *P*-values were computed. Figure 1A and B shows the results obtained in the AML-OHSU-2022 dataset, in which 97 genes were significantly downregulated and 56 genes were significantly upregulated in IDH-MUT samples. Similar analyses were performed in the remaining four datasets.

Intersection analysis of significantly deregulated genes across datasets identified three genes, CD93, LGALS3BP, and ROGDI, that were consistently downregulated in IDH-MUT samples in all five cohorts (Fig. 1C), while no common upregulated genes were observed (Fig. 1D). These three genes were therefore selected as stable IDH-associated readouts and used for downstream quality control and biological validation of predicted groups.

To assess the robustness of these findings to the choice of statistical method, we performed complementary analyses using DESeq2 on RNA-seq datasets with available raw counts (GSE106291, GSE146173, and TCGA-LAML), while retaining the Mann–Whitney U test for datasets lacking count data (AML-OHSU-2022 and GSE6891). This approach confirmed the consistent downregulation of CD93 and ROGDI across all datasets using the same significance and fold-change criteria, whereas LGALS3BP did not meet the significance threshold in two RNA-seq datasets (GSE106291 and GSE146173).

### Construction of a pooled AML compendium comprising 5844 samples

Transcriptomic profiles from 19 AML datasets were merged into a single compendium, and batch effects were corrected using pyComBat. Prior to correction, samples clustered primarily by dataset, reflecting strong technical variability. After correction, dataset-specific clustering largely disappeared, indicating effective harmonization (Supplementary Fig. S1A and B).

**Figure 2.**
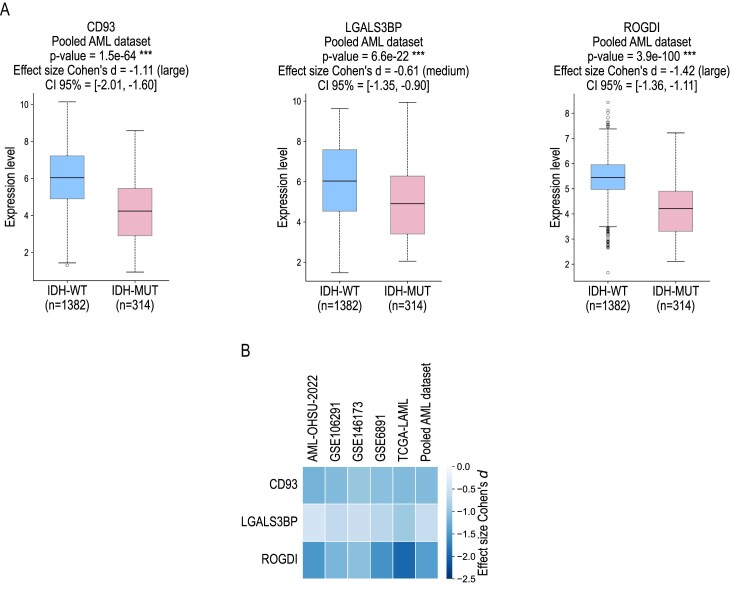
Differential expression of IDH status readout genes in AML. Expression levels of three transcriptional readout genes (CD93, LGALS3BP, and ROGDI) were compared between IDH-WT and IDH-MUT tumors. (**A**) Boxplots showing gene expression distributions for each readout gene in the pooled AML dataset. All three genes are significantly upregulated in IDH-WT compared with IDH-MUT tumors, with medium-to-large effect sizes. (**B**) Heatmap displaying Cohen’s d effect sizes for differential expression between IDH-WT and IDH-MUT tumors across five original AML datasets with available mutation annotations and in the pooled AML dataset. In all datasets, the corresponding analysis of variance (ANOVA) *P*-values were $< $.05. Absolute values of effect sizes were interpreted as negligible (d $\le$ 0.2), small (0.2 $< $ d $\le$ 0.5), medium (0.5 $< $ d $\le$ 0.8), and large (d $> $ 0.8).

To ensure preservation of biological signals, we plotted expression levels of the three readout genes relative to IDH mutation status (CD93, LGALS3BP, and ROGDI) before and after pyComBat correction (Supplementary Fig. S1C). A near-perfect correlation was observed between pre- and post-correction expression levels in all AML datasets, indicating that pyComBat correction did not alter the main biological features related to IDH status. Differential analysis in the pooled dataset confirmed significant downregulation of all three genes in IDH-MUT samples (Fig. 2A), consistent with effect sizes observed in individual AML datasets (Fig. 2B). In all cases, results remained highly similar and statistically significant, confirming that pyComBat correction did not distort the underlying biological signals in our transcriptomic data.

After harmonization, we obtained one pooled AML dataset containing expression levels of 9870 genes shared across studies and 5844 AML samples in total. In this pooled dataset, IDH mutation status was available for 1696 samples (29%), including 314 IDH-MUT (18.5%) and 1382 IDH-WT (81.5%), while 4148 samples lacked mutation annotations. Detailed gene availability across datasets and the proportion of retained genes are provided in Supplementary Table S1.

To assess the representation of genes involved in IDH-related biology within the retained 9870 genes, we examined the presence of key genes associated with epigenetic regulation, metabolism, and differentiation. Most functional pathways remained represented despite partial gene loss. In particular, epigenetic regulation was extensively covered, including ASXL1, DNMT3A, and multiple $\alpha$-ketoglutarate-dependent histone demethylases from several KDM families (KDM2–KDM6). Metabolic pathways were comprehensively represented (IDH1/2, LDHA, MDH1/2, OGDH, PHGDH), and key transcriptional programs associated with stemness and myeloid differentiation were preserved (CEBPA, MEIS1).

For model development, samples were divided into two subsets. A training subset comprising four datasets (AML-OHSU-2022, GSE106291, GSE146173, GSE6891) included 1546 samples (285 IDH-MUT and 1261 IDH-WT) and was used for nested cross-validation. The independent validation subset consisted of the TCGA-LAML cohort (29 IDH-MUT and 121 IDH-WT). Gene expression data were represented as a matrix with samples as rows and genes as columns, and IDH status was encoded as binary labels (0 $=$ WT, 1 $=$ MUT).

Two predictive models, a nonlinear NN and a regularized LR, were trained on the training subset and compared. The best-performing model was then selected, evaluated on the independent validation cohort, and subsequently retrained on all annotated samples to infer IDH status for cases lacking mutation annotations.

### NN predicts IDH mutation status with high specificity but lower sensitivity for IDH-MUT cases

We trained a feed-forward NN on the 1546 AML samples with available IDH status using nested cross-validation. Hyperparameter optimization identified an architecture with two hidden layers (64 and 16 neurons) and dropout rates of 0.3 and 0.2 as optimal. Similar architectures have been proposed in prior studies of NNs applied to transcriptomic data, where shallow architectures often exhibit stable performance across a range of hyperparameter settings [24].

Across outer test folds, the NN achieved the following performance: ROC-AUC $=$ 0.984 $\pm$ 0.005, accuracy $=$ 0.972 $\pm$ 0.006, balanced accuracy $=$ 0.941 $\pm$ 0.016, sensitivity $=$ 0.891 $\pm$ 0.034 and specificity $=$ 0.990 $\pm$ 0.006. The detailed metrics are presented in Supplementary Fig. S2. Overall, the NN obtained consistent predictive performance for IDH mutation status in AML with good discrimination between IDH-MUT and IDH-WT samples. Among 1261 confirmed IDH-WT samples, 1249 samples (99.0%) were correctly predicted and 12 samples (1.0%) were misclassified as IDH-MUT. Among 285 confirmed IDH-MUT samples, 254 samples (89.1%) were correctly identified, while 31 samples (10.9%) were misclassified as IDH-WT. Performance for the clinically relevant IDH-MUT class is characterized by a low false-positive rate together with a relatively high false-negative rate, suggesting that the model prioritizes specificity over sensitivity for mutation detection, and a subset of mutant samples remains challenging to detect. High discrimination performance of the NN model is confirmed by average and individual ROC-AUC curves obtained for each test fold. As expected, the by-class precision, recall, and f1 metrics show low variability across test folds for the IDH-WT group, and a greater variability for the IDH-MUT group. Importantly, probability calibration metrics were favorable, as reflected by a low Brier score of 0.023 $\pm$ 0.006, indicating that predicted probabilities closely matched observed outcome frequencies. A calibration curve of the NN model shows slightly underestimated probabilities in the low-range region, with an overall good calibration in the mid and high range regions.

### LR classifier outperforms the NN with both higher specificity and sensitivity for IDH-MUT cases

**Figure 3.**
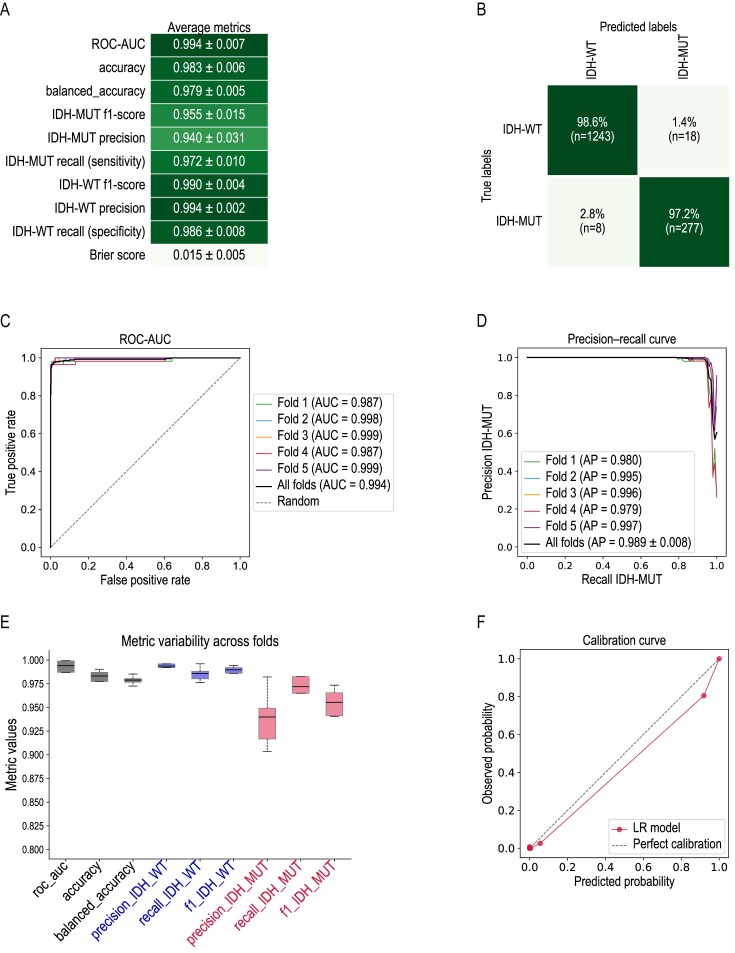
Predictive performance of the LR model. (**A**) Mean performance metrics obtained across all cross-validation test folds. (**B**) Confusion matrix comparing true and predicted labels aggregated across test folds and normalized by the true class distribution. (**C**) ROC curves for each test fold and the pooled predictions, with corresponding AUC values. The ROC curve represents the true positive rate versus the false positive rate across decision thresholds. (**D**) Precision–recall curves for each test fold and pooled predictions. Average precision corresponds to the area under the precision–recall curve. (**E**) Boxplots showing the distribution of performance metrics across test folds. (**F**) Calibration plot comparing predicted probabilities with observed outcome frequencies, computed by grouping predictions into 10 equal-frequency (quantile) bins and plotting the mean predicted probability against the observed proportion of positive cases in each bin.

Given the high performance of the NN, we next evaluated whether a simpler linear model could achieve comparable or improved results. We trained a LR classifier using hyperparameter tuning, which resulted in an optimal L2-regularized linear decision boundary with the inverse regularization strength parameter C $=$ 8, a maximum of 500 optimization iterations, and a minimum variance threshold across training folds of 0.4. This model achieved superior discrimination compared to the NN, with ROC-AUC $=$ 0.994 $\pm$ 0.007, accuracy $=$ 0.983 $\pm$ 0.006, balanced accuracy $=$ 0.979 $\pm$ 0.005, sensitivity = 0.972 $\pm$ 0.010, and specificity $=$ 0.986 $\pm$ 0.008 across test folds (Fig. 3A). Importantly, the LR provided a more balanced trade-off between false-negative and false-positive cases of 2.8% and 1.4% respectively (Fig. 3B), compared to the NN model for which 10.9% and 1.0% were obtained. As for the NN model, we observed a higher variability of precision, recall, and f1 metrics in the IDH-MUT compared to IDH-WT class, due to class imbalance (Fig. 3E). Despite a low Brier calibration score of 0.015 $\pm$ 0.005, the calibration curve of the LR model showed systematic overestimation of predicted probabilities, especially in the high-probability range (Fig. 3F).

To evaluate whether the use of mixed RNA-seq normalization units (RPKM and TPM) affected model performance, we carried out a sensitivity analysis using TPM normalization for datasets where raw data were available, as described in Supplementary Methods. The LR model with an alternative TPM-based normalization scheme achieved performance metrics nearly identical to those obtained with the original RPKM-based pipeline, with ROC-AUC = 0.994, accuracy = 0.983, and balanced accuracy = 0.978 across cross-validation folds (Supplementary Fig. S3). In addition, we compared predicted probabilities obtained from the RPKM-based and TPM-based pipelines. Predictions were highly concordant, with a Pearson correlation coefficient of 0.9954. Only three samples showed discordant classification between the two approaches, with an overall agreement of 99.81% (Supplementary Fig. S4), demonstrating that the predictive framework is robust to differences in RNA-seq normalization.

To evaluate the dependence of model performance on a specific gene set, we performed a robustness analysis based on repeated random subsampling of gene features. Predictive performance remained stable across a wide range of gene subset sizes (Supplementary Fig. S5), indicating that classification is driven by broader transcriptomic signals rather than a specific subset of genes.

We also examined the genes with the highest absolute coefficients in the LR model, corresponding to the strongest contributors to prediction. Over-representation analysis of these genes did not identify significantly enriched pathways after multiple testing correction. However, annotation of the top-ranked genes indicated their involvement in biological processes relevant to AML, including epigenetic regulation, cellular metabolism, hematopoietic differentiation, and immune signaling. The list of LR coefficients is provided in Supplementary Table S2.

Overall, these results demonstrate that a carefully tuned LR model exceeded the performances in discrimination of a more complex nonlinear NN architecture, while offering important advantages in transparency, stability, interpretability and ease of deployment. However, in terms of calibration, the LR model overestimated predicted probabilities, indicating that the LR model is particularly performant for IDH status prediction but the obtained prediction probabilities should not be directly interpreted, specifically, they should not be used in terms of likelihood of IDH-MUT status.

Based on the average performance metrics calculated for the NN and LR models, we finally selected the LR model as our best model for an independent validation in the TCGA-LAML dataset and for subsequent IDH status predictions in new AML samples for which IDH status was unknown.

### Independent validation in the TCGA-LAML cohort confirms high predictive accuracy

**Figure 4.**
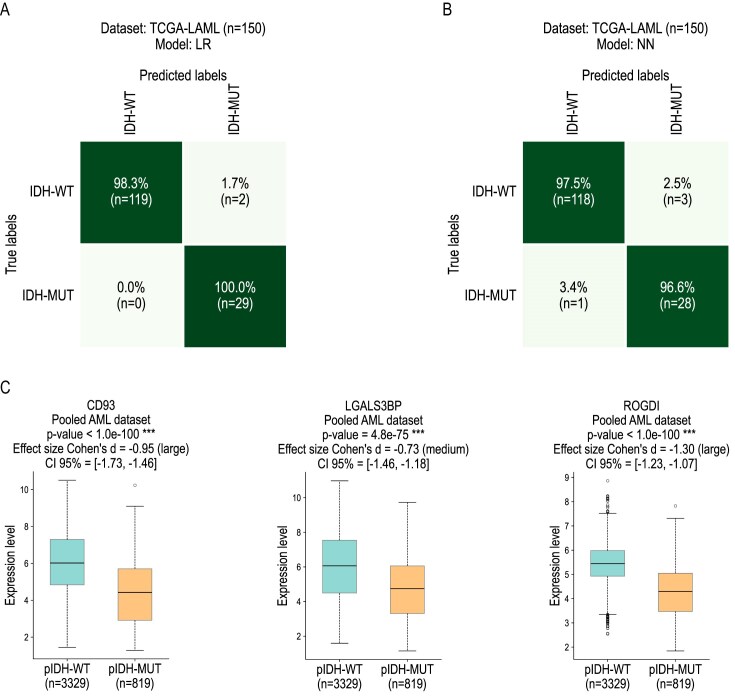
Independent validation of IDH status prediction in the TCGA-LAML cohort and evaluation of the prediction consistency in the pooled AML dataset. (**A**) Confusion matrix comparing true and predicted labels for the LR model. (**B**) Confusion matrix comparing true and predicted labels for the NN model. (**C**) Boxplots of three transcriptional readout genes (CD93, LGALS3BP, and ROGDI) comparing predicted IDH-wildtype (pIDH-WT) and predicted IDH-mutant (pIDH-MUT) tumors in the pooled AML dataset. Asterisks denote levels of ANOVA test significance: *P <.05, **P <.01, ***P <.001. Absolute values of Cohen’s effect sizes d were interpreted as negligible (d $\le$ 0.2), small (0.2 $< $ d $\le$ 0.5), medium (0.5 $< $ d $\le$ 0.8), and large (d $> $ 0.8).

We evaluated the selected LR model on the independent TCGA-LAML cohort. Predicted IDH status for 150 AML samples of the TCGA-LAML dataset was compared to their actual IDH status, using a confusion matrix normalized on true labels (Fig. 4A). All 29 IDH-MUT samples were correctly identified by the LR model. Among 121 IDH-WT samples, 119 (98.3%) were correctly classified, with two false positives.

For comparison, we additionally calculated predictions of the NN model (Fig. 4B). As expected, NN produced slightly lower performances than the LR classifier. In total, the NN model misclassified four samples: one sample (3.4%) was incorrectly predicted as IDH-WT among 29 confirmed IDH-MUT samples and, inversely, three samples (2.5%) were misclassified as IDH-MUT among 121 confirmed IDH-WT samples.

To quantify uncertainty associated with the limited sample size, 95% CIs were computed for all validation metrics. The following results were obtained: sensitivity (29/29) 95% CI [0.881–1.000], specificity (119/121) 95% CI [0.942–0.998], accuracy 95% CI [0.967–1.000], balanced accuracy 95% CI [0.979–1.000], and ROC-AUC 95% CI [0.979–1.000].

In the primary analysis, batch correction parameters were estimated jointly across all datasets to construct a harmonized compendium. Importantly, this step is unsupervised and does not use class labels. However, to address potential information leakage arising from batch correction, we performed an additional sensitivity analysis in which the batch correction was repeated using parameters estimated exclusively on the four training datasets (AML-OHSU-2022, GSE106291, GSE146173, and GSE6891), which were then applied to the independent TCGA-LAML cohort to ensure strict separation between training and validation data. In this setting, 118 out of 121 IDH-WT samples were correctly classified (97.5%), with three misclassifications, while all 29 IDH-MUT samples were correctly identified (100% sensitivity), indicating the robustness of model performance to the preprocessing strategy.

These results successfully confirmed the high discriminatory performance of the LR model for IDH status prediction. Therefore, it was used for reconstruction of IDH mutation status in previously unannotated AML samples.

### Reconstruction of IDH mutation status across public AML transcriptomic datasets

We retrained our best LR model using all 1696 samples with known IDH status and applied it to the remaining 4148 samples lacking annotations. The distributions of IDH-WT and IDH-MUT samples in the individual and pooled AML datasets are shown in Supplementary Fig. S6. Predicted labels were denoted pIDH-MUT and pIDH-WT. The LR model pIDH-MUT status for 819 tumors (19.7%) out of 4148 samples with initially unknown IDH status, and IDH-WT status for the remaining 3329 samples (80.6%). Detailed predictions for each dataset are provided in Supplementary Table S3.

### Predicted IDH groups recapitulate known biological pathways

To assess the biological consistency of the predictions, we performed ANOVA tests for the three readout genes between the predicted pIDH-WT and pIDH-MUT tumors in the pooled AML dataset (Fig. 4C), and compared them to the previously obtained results for the same genes between the confirmed IDH-WT and IDH-MUT groups, presented in Fig. 2A. A significant downregulation of all three genes, CD93, LGALS3BP, and ROGDI, with medium to large Cohen effect sizes was found in the pIDH-MUT versus pIDH-WT group, indicating that biological significance was correctly captured by the LR predictive model.

To further confirm the conservation of biological pathways between the groups of tumors with confirmed and predicted IDH status, we additionally performed several GSEA. The GSEA analysis developed by the Broad Institute [25, 26] determines whether an *a priori* defined set of genes shows statistically significant differences between two biological states. This method focuses on gene sets that share common biological functions. More than 10 000 published gene sets are available for GSEA analysis in several collections of the MSigDB database [27]. For our analysis we selected the H (hallmarks), C2 (curated gene sets), and C5 (ontology gene sets) collections.

We conducted the GSEA analyses in two different contexts: first, for the confirmed and, second, for the predicted IDH-related tumors. In the first experiment, we performed differential expression analyses between the confirmed IDH-MUT versus IDH-WT tumors separately in five AML datasets with available IDH status, as well as in the pooled AML dataset. For all genes of the genome, we calculated the corresponding fold changes of the average expression levels between the two compared groups. We then ordered all the genes by the obtained fold change values, from highest to lowest, and performed the subsequent GSEA analyses to identify significantly enriched or depleted gene sets for which the obtained nominal *P*-value <.05 and FDR $< $ 0.25.

**Figure 5.**
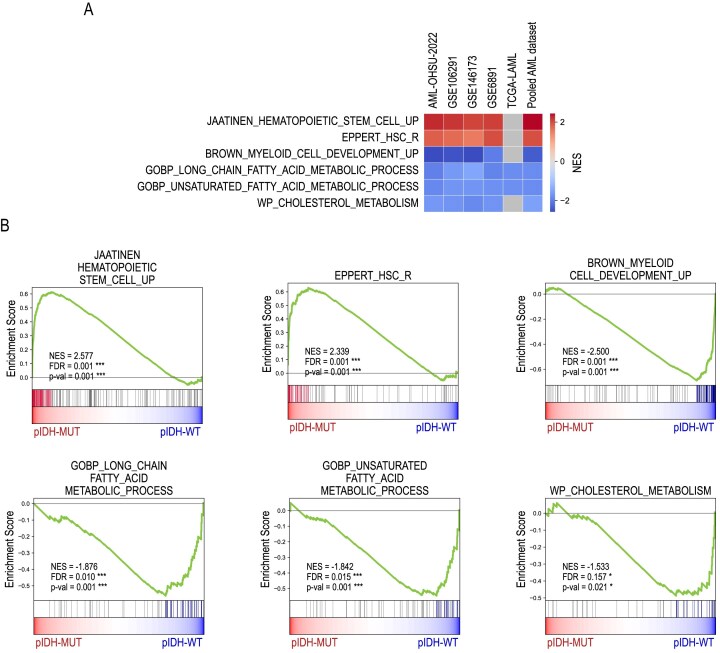
GSEA for confirmed and predicted IDH status groups. (**A**) Heatmap of normalized enrichment scores (NES) for six selected gene sets related to hematopoietic stem cell programs, myeloid differentiation, and fatty acid/cholesterol metabolism, comparing confirmed IDH-MUT versus IDH-WT tumors in five original datasets and the pooled AML dataset. Gray cells indicate nonsignificant results. (**B**) GSEA enrichment plots for the same six gene sets comparing pIDH-MUT versus pIDH-WT tumors in the pooled AML dataset. Positive NES indicates enrichment, negative NES indicates depletion. Significance was defined as nominal *P* <.05 and FDR $< $ 0.25, with asterisks denoting levels of significance: *P <.05 or FDR $< $ 0.25, ** *P* <.01 or FDR $< $ 0.1, ***P <.001 or FDR $< $ 0.05.

Figure 5A shows a selection of gene sets that were found significant in at least four datasets out of five, and for which the corresponding biological mechanisms have already been described in the literature related to an IDH mutation context in AML. Specifically, we found a significant enrichment for two molecular signatures of hematopoietic stem cells, alongside with a significant depletion of myeloid cell development signature, corresponding to known mechanisms of hematopoietic differentiation blockade and stemness in IDH-mutation context [4, 28, 29]. Interestingly, we also observed a consistent and significant depletion of three molecular signatures related to fatty acid and cholesterol metabolism. Indeed, under physiological conditions, IDH enzymes contribute to cellular lipid metabolism and mitochondrial function through NADPH generation [30–32], while in IDH-MUT AML context, in concordance with our results, a reduction in fatty acid metabolites was reported in a comprehensive metabolomic study of primary AML blasts [33].

A second GSEA comparing predicted pIDH-MUT and pIDH-WT groups in the pooled AML dataset confirmed the enrichment or depletion of all six pathways (Fig. 5B), demonstrating that the LR-based predictions preserved biologically meaningful transcriptional programs.

To assess the impact of restricting the compendium to genes shared across datasets, we quantified the proportion of genes retained within each gene set used for GSEA. Across the selected gene sets, 62%–71% of genes were retained in the pooled AML dataset of 9870 genes (Supplementary Table S4). This level of coverage indicates that the majority of pathway components were preserved, supporting the robustness of enrichment analyses despite restriction to shared genes.

## Discussion

In this study, we developed2 and systematically evaluated two machine learning approaches, a feed-forward NN and a regularized LR model, for predicting IDH mutation status in AML from transcriptomic profiles. Using large, heterogeneous gene expression datasets and rigorous nested cross-validation, we demonstrate that IDH mutation status can be accurately inferred from gene expression alone, without requiring direct genomic sequencing. While both models achieved excellent discrimination performance, with average ROC-AUC values exceeding 0.98, the LR model consistently outperformed the NN in terms of balanced accuracy, sensitivity for IDH-MUT cases, and external validation robustness. Despite its linear nature, LR was sufficient to capture the dominant predictive structure present in the data. Notably, LR achieved a more balanced trade-off between sensitivity and specificity compared to NN, substantially reducing false-negative predictions for IDH-MUT cases (2.8% versus 10.9%).

Calibration analyses further1 highlighted complementary strengths. The NN produced visually well-aligned calibration curves and a low Brier score of 0.023, indicating reliable probability estimates. LR achieved an even lower Brier score of 0.015 but showed systematic overestimation at high predicted probabilities, underscoring the distinction between discrimination and probability calibration. While this does not affect its suitability for binary classification, *post hoc* calibration would be advisable if probability outputs are used quantitatively, for example in probabilistic modeling or integrative risk frameworks. Importantly, the interpretability and reproducibility of LR, together with its superior external performance, motivated its selection as the final model.

The strong generalization performance of the LR model was further confirmed through independent validation on the TCGA-LAML dataset, which was entirely excluded from model training and tuning. The LR model correctly identified all 29 IDH-MUT samples in TCGA-LAML and misclassified only 2 out of 121 IDH-WT cases, demonstrating excellent robustness across platforms and cohorts. Based on the obtained performances, the LR model was finally selected for large-scale IDH status reconstruction in public AML cohorts. We successfully predicted IDH mutation status for 4148 AML samples from transcriptomic data, including 819 IDH-MUT and 3329 IDH-WT tumors. This large-scale reconstruction creates a valuable resource for investigating transcriptional programs associated with IDH mutations and enables analyses that would otherwise be limited by incomplete genomic data.

Accurate inference of IDH mutation status from gene expression data has important implications for AML research, particularly in retrospective studies and meta-analyses where genomic mutation data may be unavailable. The ability to reconstruct IDH status across thousands of samples enables the expanded investigation of IDH-associated biology, transcriptional programs, and clinical correlations at an unprecedented scale. Moreover, this approach may facilitate hypothesis generation regarding IDH-driven leukemogenesis and treatment response in cohorts lacking comprehensive mutational profiling.

Interestingly, three genes, CD93, LGALS3BP, and ROGDI, were found consistently downregulated in IDH-MUT samples across all AML datasets with available IDH status. CD93 and LGALS3BP share functional roles in extracellular matrix interaction, cell adhesion, and modulation of the tumor microenvironment. Both proteins operate at the cell surface or in the extracellular space and are involved in interactions between leukemic cells and stromal, endothelial, and immune components. They have also been linked to inflammatory signaling and angiogenesis, suggesting that their co-regulation reflects a coordinated program of microenvironmental adaptation [34, 35]. In contrast, ROGDI is poorly characterized in AML, but its consistent downregulation may reflect a common regulatory layer associated with IDH-driven epigenetic reprogramming.

However, some limitations should be acknowledged. First, although extensive cross-validation and independent testing were performed, the study remains retrospective and relies on publicly available datasets with varying platforms and pre-processing pipelines. Second, the observed probability miscalibration of the LR model suggests that further refinement may be required for applications involving probabilistic risk estimation. Another limitation is the class imbalance between IDH-WT and IDH-MUT samples, reflecting the known prevalence of IDH mutations in AML. Although class imbalance was explicitly addressed using stratified nested cross-validation, class weight adjustments, and balanced metrics, performance estimates for the minority class showed greater variability across cross-validation folds, suggesting that the limited number of IDH-MUT samples may not fully capture the transcriptional heterogeneity of this subgroup. Future studies incorporating larger numbers of IDH-MUT cases or complementary data modalities may further improve sensitivity and robustness for minority-class prediction. Additionally, systematic analysis of the genes driving LR predictions may yield novel insights into transcriptional consequences of IDH mutations and their interaction with broader AML molecular subtypes. In line with this perspective, analysis of LR model coefficients suggests that predictions are not driven by a small number of highly specific markers, but rather by the combined contribution of many genes with moderate effects. This broad structure likely reflects the global transcriptional reprogramming induced by IDH mutations, which is mediated by widespread epigenetic and metabolic alterations linked to 2-hydroxyglutarate accumulation.

The integration of RNA-seq datasets processed using different normalization strategies, including RPKM and TPM, may introduce systematic distributional shifts that are only partially mitigated by batch correction. Nevertheless, multiple analyses support the robustness of our approach. PCA showed effective removal of dataset-specific structure after correction, while expression levels of IDH-associated readout genes remained highly concordant before and after correction, indicating preservation of biological signals. Consistent differential expression patterns in the pooled dataset further support this conclusion. In addition, the strong predictive performance and sensitivity analyses demonstrating consistent results across normalization schemes suggest that residual variability did not substantially affect the biological signal captured by the model.

The independent validation cohort (TCGA-LAML) included a limited number of IDH-MUT samples (*n* = 29), which constrains the precision of performance estimates, particularly for sensitivity, as reflected by relatively wide CIs. Furthermore, in the primary analysis, batch correction was performed jointly across datasets, which may introduce a degree of information sharing at the preprocessing stage. However, sensitivity analyses using strictly independent batch correction yielded consistent results, supporting the robustness of our findings.

An important consideration for practical application concerns the use of the model on new, independently generated samples. In this study, transcriptomic data were harmonized across cohorts using pyComBat, which requires multiple samples to estimate batch-specific parameters and is therefore not directly applicable to single-sample inputs. Consequently, our framework is primarily designed for cohort-level analyses, such as reconstruction of missing IDH annotations in public datasets, where multiple samples are available for joint normalization and batch-effect correction. For new data, two strategies are possible. One practical approach is to integrate new samples with a reference compendium, perform batch correction using dataset-of-origin as a batch variable, and then apply the trained model using identical preprocessing, scaling, and hyperparameter tuning. Alternatively, a more rigorous approach is to recompute normalization and batch-effect correction jointly across the original and new datasets. While differences in upstream bioinformatics pipelines (e.g. alignment, quantification, or normalization methods) may introduce variability, the strong performance observed across 19 heterogeneous datasets suggests that the model is robust to typical inter-study differences. Nevertheless, performance may be affected in cases of substantial technical divergence or when only a very limited number of samples are available for normalization [36, 37].

While our model demonstrates high accuracy in inferring IDH mutation status from transcriptomic data, this approach is not intended for clinical diagnostic use. Clinical determination of IDH mutations continues to rely on established molecular assays, which remain the gold standard. Instead, our framework addresses a critical research need: reconstructing missing IDH annotations in public AML transcriptomic datasets. By expanding the number of molecularly characterized samples, this approach enables large-scale integrative analyses, including meta-analyses, biomarker discovery, pathway enrichment studies, mechanistic investigations of IDH-driven transcriptional, and metabolic programs.

Another aspect concerns the small subset of misclassified samples, which may arise from both technical and biological sources. From a technical perspective, residual batch effects, differences in sequencing depth, platform-specific biases, or variability in sample quality (e.g. RNA degradation, blast percentage, or tumor purity) could attenuate transcriptional signals and shift samples toward the incorrect class. From a biological standpoint, discordance between genotype and transcriptomic phenotype may also contribute. In particular, some IDH-WT cases predicted as mutant may reflect metabolic states characterized by elevated 2-HG independent of canonical IDH mutations. Indeed, several enzymes, including malate dehydrogenase, lactate dehydrogenase A, and phosphoglycerate dehydrogenase, can promiscuously reduce $\alpha$-ketoglutarate to 2-HG under specific metabolic conditions, suggesting that activation of alternative 2-HG–producing pathways could generate an IDH-MUT-like transcriptional program [38]. Conversely, IDH-MUT samples misclassified as WT may correspond to cases in which intracellular 2-HG levels are comparatively low, potentially due to enhanced degradation or activation of 2-HG clearance pathways, thereby attenuating the downstream epigenetic and transcriptional footprint of the mutation. These hypotheses highlight that misclassification may reflect biologically informative states rather than purely model errors and warrant further investigation using integrated metabolomic or functional analyses.

Although IDH mutations also occur in other malignancies such as glioma, the present model is not designed for cross-tissue application. Fundamental differences in cellular origin, baseline transcriptional programs, and downstream effects of IDH mutations limit the transferability of gene expression-based predictors across cancer types. The development of pan-cancer predictive models represents an interesting direction for future research but would require dedicated methodological frameworks as well as careful tissue-specific calibration.

In conclusion, we demonstrated that IDH mutation status in AML can be accurately predicted from transcriptomic data using machine learning. A carefully tuned LR model outperformed a more complex NN architecture, achieving superior discrimination, robustness, and interpretability. Validation on an independent cohort and biologically consistent reconstruction of IDH status in thousands of AML samples highlight the utility of this approach for large-scale transcriptomic studies and translational research in AML.

## Supplementary Material

lqag058_Supplemental_Files

## Data Availability

The datasets analyzed in this study are publicly available, with accession numbers and identifiers listed in Table 1. Predicted IDH mutation labels for each dataset, generated using our logistic regression model, are provided in Supplementary Table S1. The machine-learning models, implemented in Python, and batch-corrected gene expression matrix obtained by integrating 19 AML transcriptomic cohorts using pyComBat, are available in the Zenodo repository https://zenodo.org/records/19429975 (DOI: 10.5281/zenodo.19429975). All additional data outputs supporting the conclusions of this article are included within the manuscript and its supplementary files.
