# Supplementary material for: Predicting isocitrate dehydrogenase mutation status in acute myeloid leukemia from gene expression profiles by machine learning
Source: NAR Genom Bioinform. 2026 Jun 8;8(2):lqag058. doi: 10.1093/nargab/lqag058 (PMC13244157; doi:10.1093/nargab/lqag058)
Supplement: lqag058_Supplemental_Files [file lqag058_supplemental_files.zip › Jung_et_al_Supplementary_data.pdf]

Predicting isocitrate dehydrogenase mutation status in acute myeloid leukemia from gene expression profiles by machine learning

Ina Jung, Anne-Laure Vitte, Florent Chuffart, Simon Chevalier, Pascal Mossuz, Saadi Khochbin and Ekaterina Bourova-Flin

Supplementary data

Contents

1 Supplementary Methods **2**

1.1 Sensitivity analysis of RNA-seq normalization strategies 2

1.2 Neural network architecture 2

2 Supplementary Tables **4**

3 Supplementary Figures **6**

1 Supplementary Methods

1.1 Sensitivity analysis of RNA-seq normalization strategies

To assess the impact of RNA-seq normalization heterogeneity on model performance, we conducted a sensitivity analysis using transcript per million (TPM) normalization wherever possible. Specifically, TPM values were recomputed and log-transformed for RNA-seq datasets with available raw counts and IDH status annotations (GSE106291, GSE146173, and TCGA-LAML), while datasets lacking raw data (AML-OHSU-2022) or corresponding to microarray platforms (GSE6891) were retained as originally processed. Using this alternative normalization scheme, we reconstructed the training and validation framework exactly as in the primary analysis. Four datasets (GSE106291, GSE146173, GSE6891, and AML-OHSU-2022) were used for model training and nested cross-validation, while the TCGA-LAML dataset processed in TPM units was reserved for independent validation. All preprocessing steps, feature standardization, hyperparameter tuning, and evaluation metrics were kept identical to the original LR pipeline. Model performance obtained under this TPM-based framework was compared to that of the original RPKM-based pipeline. Additionally, concordance between predicted probabilities generated by the two approaches was assessed using Pearson correlation and classification agreement metrics.

1.2 Neural network architecture

A fully connected feed-forward neural network (multilayer perceptron, MLP) was implemented using PyTorch to predict IDH mutation status from gene expression profiles. MLPs were selected as a principled baseline model for transcriptomic classification tasks, as they are well suited to learning non-linear relationships in high-dimensional molecular data without imposing spatial or sequential structure on the input features [1, 2]. The network architecture consisted of an input layer matching the dimensionality of the gene expression features, followed by two hidden layers comprising 64 and 16 neurons, respectively. This progressively narrowing architecture was chosen to allow the model to first capture broad gene-level interactions and subsequently compress them into a lower-dimensional representation relevant for classification, a design commonly adopted in neural networks for omics data to balance representational capacity and regularization [3, 4]. Rectified Linear Unit (ReLU) activation functions were applied after each hidden layer to introduce non-linearity. Dropout regularization was used to mitigate overfitting, with dropout probabilities of 0.3 after the first hidden layer and 0.2 after the second hidden layer, consistent with standard practices for high-dimensional gene expression models [5, 6]. The output layer was composed of a single neuron with a sigmoid activation function, producing a probability estimate for the IDH-mutant class.

Hyperparameter tuning. Hyperparameter optimization was performed using a nested cross-validation framework to ensure unbiased performance estimation and to prevent information leakage [7]. The outer loop comprised five stratified folds and was used to assess model generalization, while the inner loop was dedicated to hyperparameter selection. Within each outer training fold, a grid search strategy was employed to explore combinations of architectural and optimization parameters, including dropout probabilities, batch size, learning rate, weight decay coefficient, and the maximum number of training epochs. The optimal hyperparameter configuration was selected based on the highest mean area under the receiver operating characteristic curve (ROC-AUC) across inner folds. The explored search space included neural networks with two hidden layers, the dropout probability of 0.3 for the first hidden layer and a range of dropout

probabilities from 0.0 to 0.3 for the second hidden layer, batch sizes of 32 or 64, learning rates and weight decay coefficients of 10^{-4} or 10^{-5}, and a maximum of 100 or 200 training epochs.

Model training and class imbalance handling. Gene expression features were standardized using z-score normalization (mean = 0, standard deviation = 1) within each training fold. Scaling parameters were estimated exclusively on training data and applied to validation and test subsets. Per-gene z-score normalization removes absolute expression levels and modifies inter-gene relationships within individual samples, which may carry biological information. However, it preserves between-sample variation for each gene, which constitutes the primary signal exploited by the models. In the context of multi-cohort integration, where residual technical variability may persist despite batch correction, standardization further ensures that all genes contribute on a comparable scale and improves model generalization across datasets.

Model training was performed using the Adam optimizer with a learning rate of 10^{-4}, an L2 weight decay coefficient of 10^{-5}, a batch size of 32 samples, and a maximum of 200 epochs. Binary cross-entropy with logits was used as the loss function. Early stopping was implemented using a stratified internal validation split (10% of training data) with a patience of 20 epochs. Given a 4.4-fold imbalance between IDH-WT and IDH-MUT samples, a weighted loss function was used. Class weights were computed within each training fold as the ratio of IDH-WT to IDH-MUT samples.

Performance evaluation. Model performance was evaluated using five-fold stratified cross-validation. In each fold, the trained model was applied to a held-out test set that was not used during training, hyperparameter tuning, or early stopping. Performance metrics included ROC-AUC, accuracy, and balanced accuracy which is defined as the average of the true positive rate (TPR) and the true negative rate (TNR). Class-specific precision, recall and F1-score metrics were reported for IDH-mutant and IDH-wildtype samples. Additionally, we calculated the Brier calibration score which measures the mean squared difference between predicted probabilities and actual outcomes. After completion of cross-validation and hyperparameter selection, a final model was trained on the entire dataset with selected hyperparameters. The trained model was then used to generate probabilistic predictions for unseen samples, which were converted into binary IDH status with a classification threshold of 0.5.

2 Supplementary Tables

Dataset	Available IDH status	Total number of genes	Coverage
AML-OHSU-2022	yes	18130	54.44%
BEATAML-1.0		38428	25.68%
GSE106291	yes	20746	47.58%
GSE111678		21890	45.09%
GSE1159		13077	75.48%
GSE13159		21875	45.12%
GSE146173	yes	23665	41.71%
GSE165430		37495	26.32%
GSE17855		21878	45.11%
GSE216738		37162	26.56%
GSE22845		21897	45.07%
GSE232130		37389	26.40%
GSE253086		38625	25.55%
GSE297413		25660	38.46%
GSE37642		21875	45.12%
GSE43176		13085	75.43%
GSE61804		21883	45.10%
GSE6891	yes	22724	43.43%
TCGA-LAML	yes	38428	25.68%

Supplementary Table 1: Gene availability across datasets. The table shows the total number of available genes in each dataset and the percentage of these genes (Coverage) included in the harmonized compendium containing 9870 genes shared across all 19 cohorts.

Excel file available online

Supplementary Table 2: Coefficients of the logistic regression (LR) model.

Excel file available online

Supplementary Table 3: Confirmed IDH-WT and IDH-MUT labels (with known annotations) and predicted labels (pIDH-WT and pIDH-MUT) assigned by the logistic regression (LR) model to previously unannotated samples in 19 AML datasets.

Gene set	Total	Matched	Coverage
BROWN_MYELOID_CELL_DEVELOPMENT_UP	167	120	71.86%
EPPERT_HSC_R	127	91	71.65%
GOBP_LONG_CHAIN_FATTY_ACID_METABOLIC_PROCESS	109	68	62.39%
GOBP_UNSATURATED_FATTY_ACID_METABOLIC_PROCESS	115	73	63.48%
JAATINEN_HEMATOPOIETIC_STEM_CELL_UP	323	216	66.87%
WP_CHOLESTEROL_METABOLISM	72	49	68.06%

Supplementary Table 4: Gene set coverage after gene intersection across AML datasets. This table quantifies pathway-level gene coverage following restriction to the 9870 genes shared across all datasets. For each gene set used in the Gene Set Enrichment Analysis (GSEA), the total number of genes in the original set (Total), the number of genes retained in the pooled AML compendium after intersection across datasets (Matched), and the corresponding proportion of retained genes (Coverage) are reported.

3 Supplementary Figures

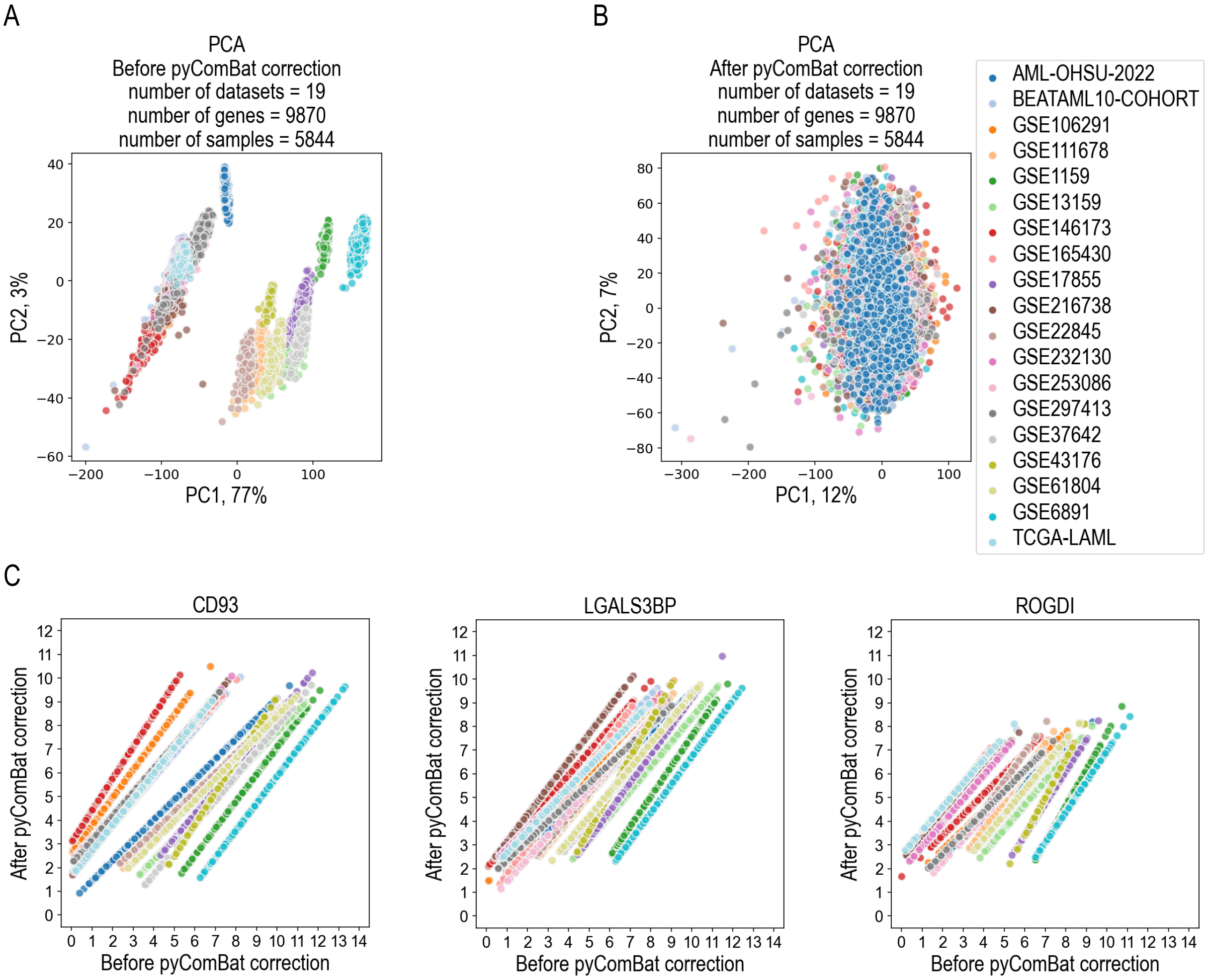

Supplementary Figure 1: Batch effect correction across 19 AML transcriptomic datasets using the pyComBat algorithm. **A**: Principal component analysis (PCA) projection of samples before batch correction, colored by dataset, illustrating dataset-specific clustering. **B**: PCA projection after batch correction, showing improved mixing of samples across datasets while preserving biological variability. **C**: Correlation plots comparing gene expression levels before and after correction for three IDH status-associated readout genes (CD93, LGALS3BP and ROGDI), demonstrating preservation of biologically relevant signals following batch adjustment.

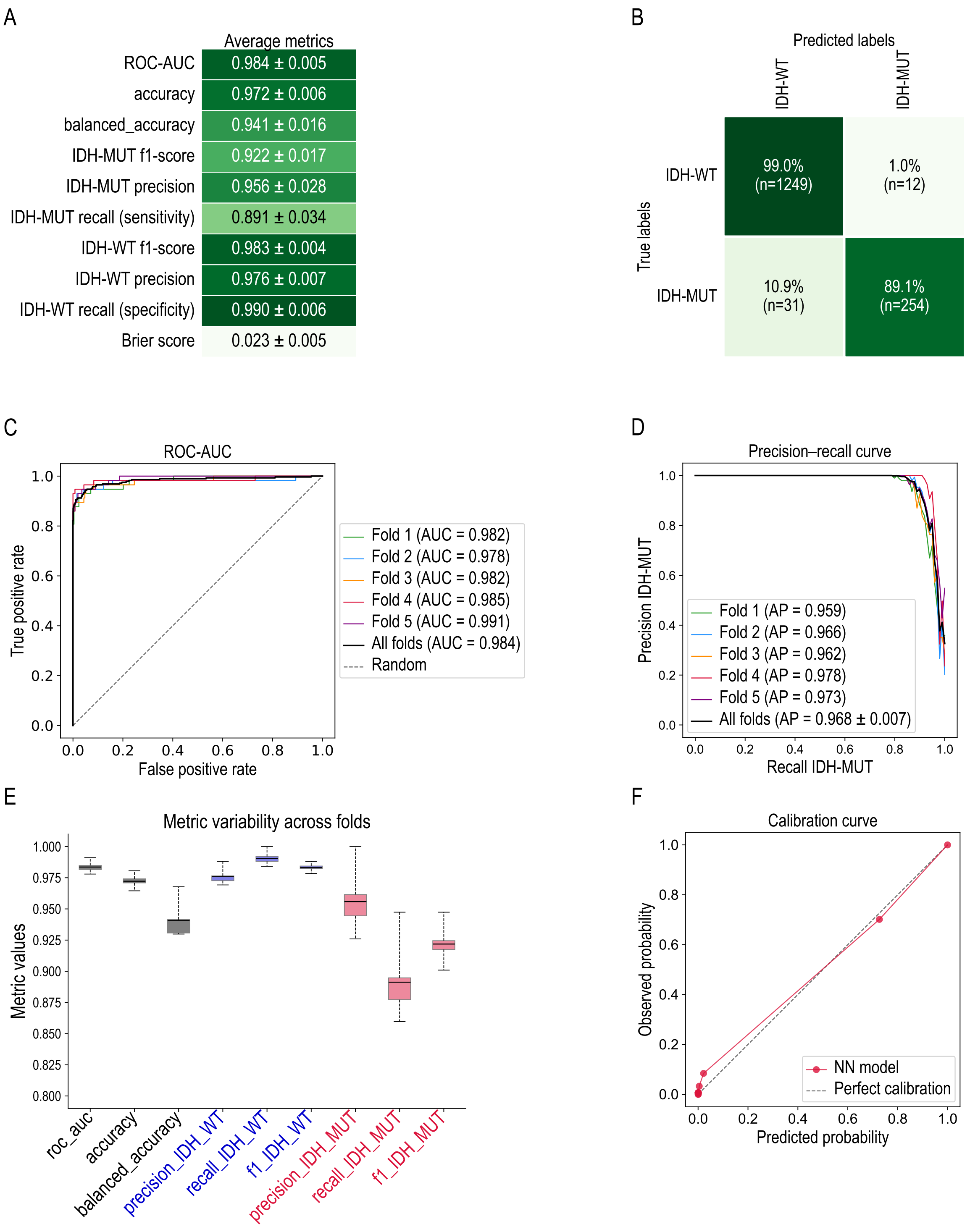

Supplementary Figure 2: Predictive performance of the neural network (NN) model. **A**: Mean performance metrics obtained across all cross-validation test folds. **B**: Confusion matrix comparing true and predicted labels aggregated across test folds and normalized by the true class distribution. **C**: Receiver operating characteristic (ROC) curves for each test fold and the pooled predictions, with corresponding area under the curve (AUC) values. The ROC curve represents the true positive rate versus the false positive rate across decision thresholds. **D**: Precision–recall curves for each test fold and pooled predictions. Average precision (AP) corresponds to the area under the precision–recall curve. **E**: Boxplots showing the distribution of performance metrics across test folds. **F**: Calibration plot comparing predicted probabilities with observed outcome frequencies, computed by grouping predictions into 10 equal-frequency (quantile) bins and plotting the mean predicted probability against the observed proportion of positive cases in each bin.

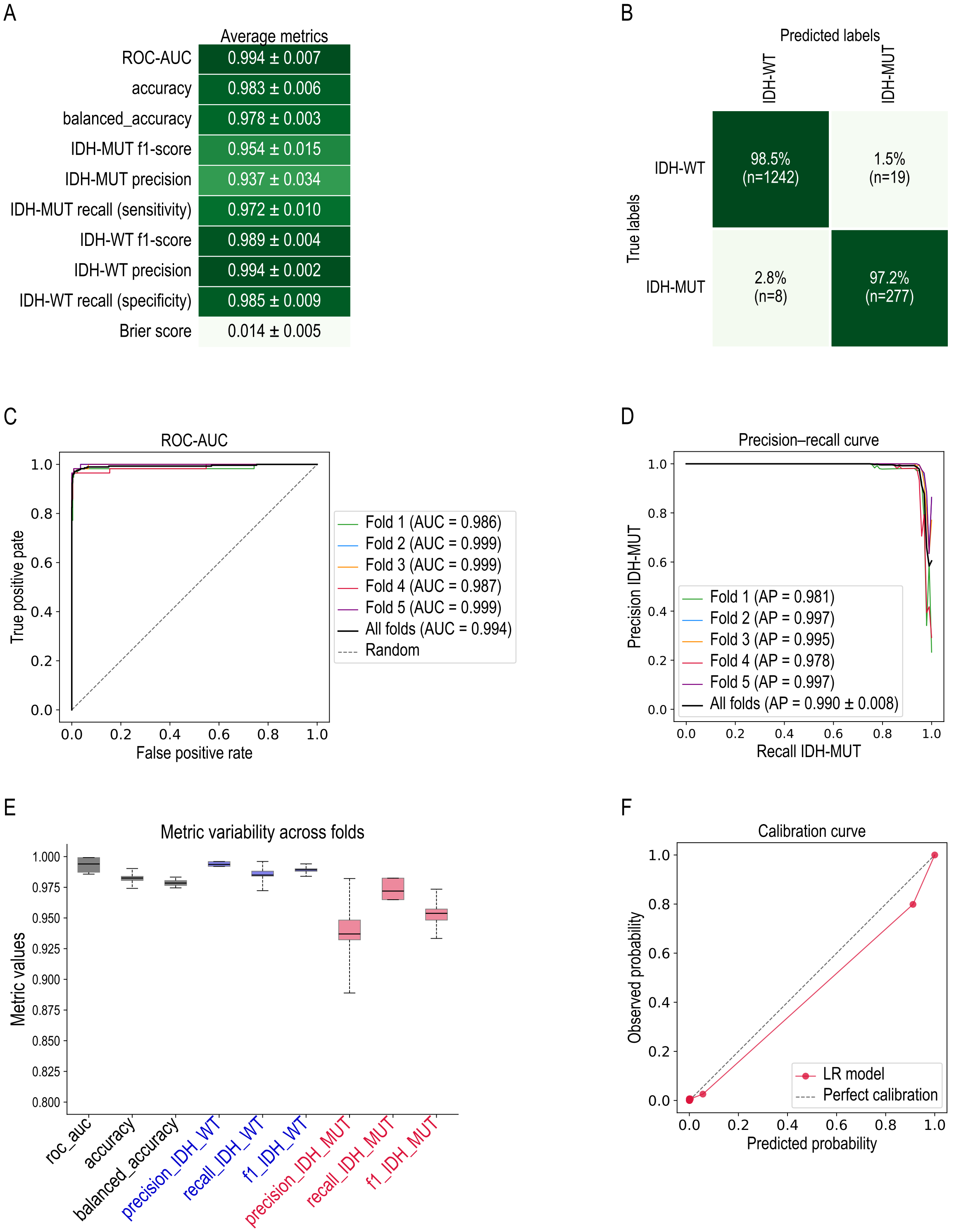

Supplementary Figure 3: Predictive performance of the logistic regression (LR) model using an alternative TPM-based normalization scheme. Panels and metrics are presented as described in Supplementary Figure 2 (mean cross-validation metrics, confusion matrix, ROC and precision–recall curves, metric distributions, and calibration plot).

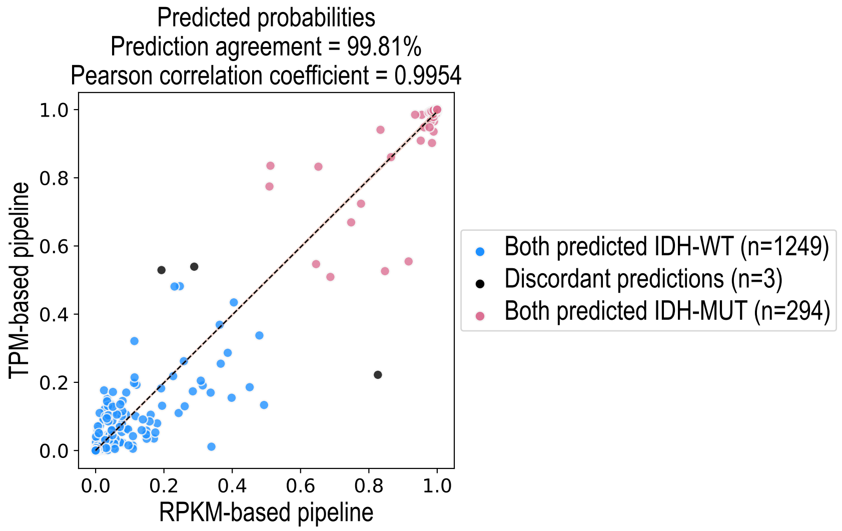

Supplementary Figure 4: Predicted probabilities calculated by the logistic regression (LR) model from the RPKM-based and TPM-based pipelines.

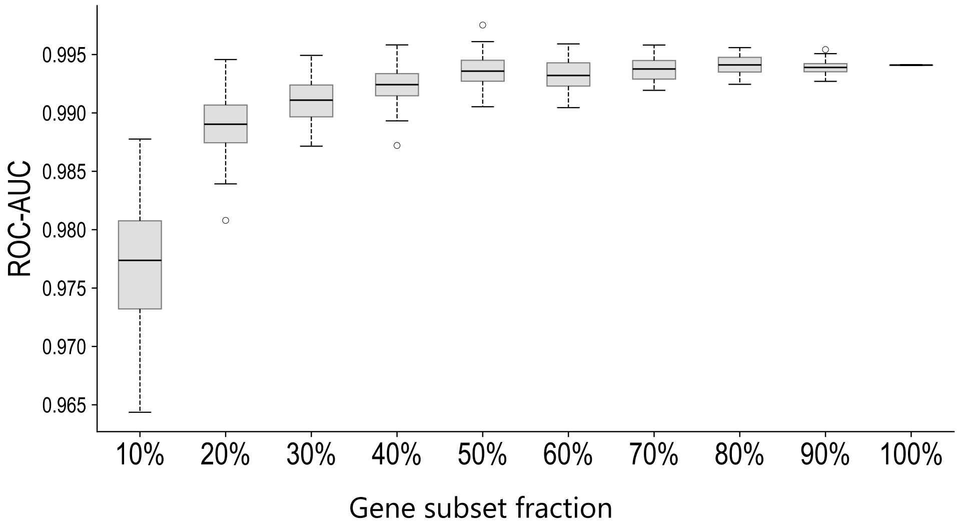

Supplementary Figure 5: Robustness of IDH mutation prediction to gene subsampling. For each subset fraction ranging from 10% to 100%, a random subset of genes corresponding to the given fraction of the total 9870 genes was selected. For each fraction, this procedure was repeated 50 times, generating 50 independent gene subsets. For every subset, the full cross-validation pipeline (five-fold stratified cross-validation using the LR model with fixed hyperparameters) was applied to the pooled AML dataset, and the corresponding ROC-AUC was computed on held-out test folds. The resulting distributions of ROC-AUC values are shown as boxplots.

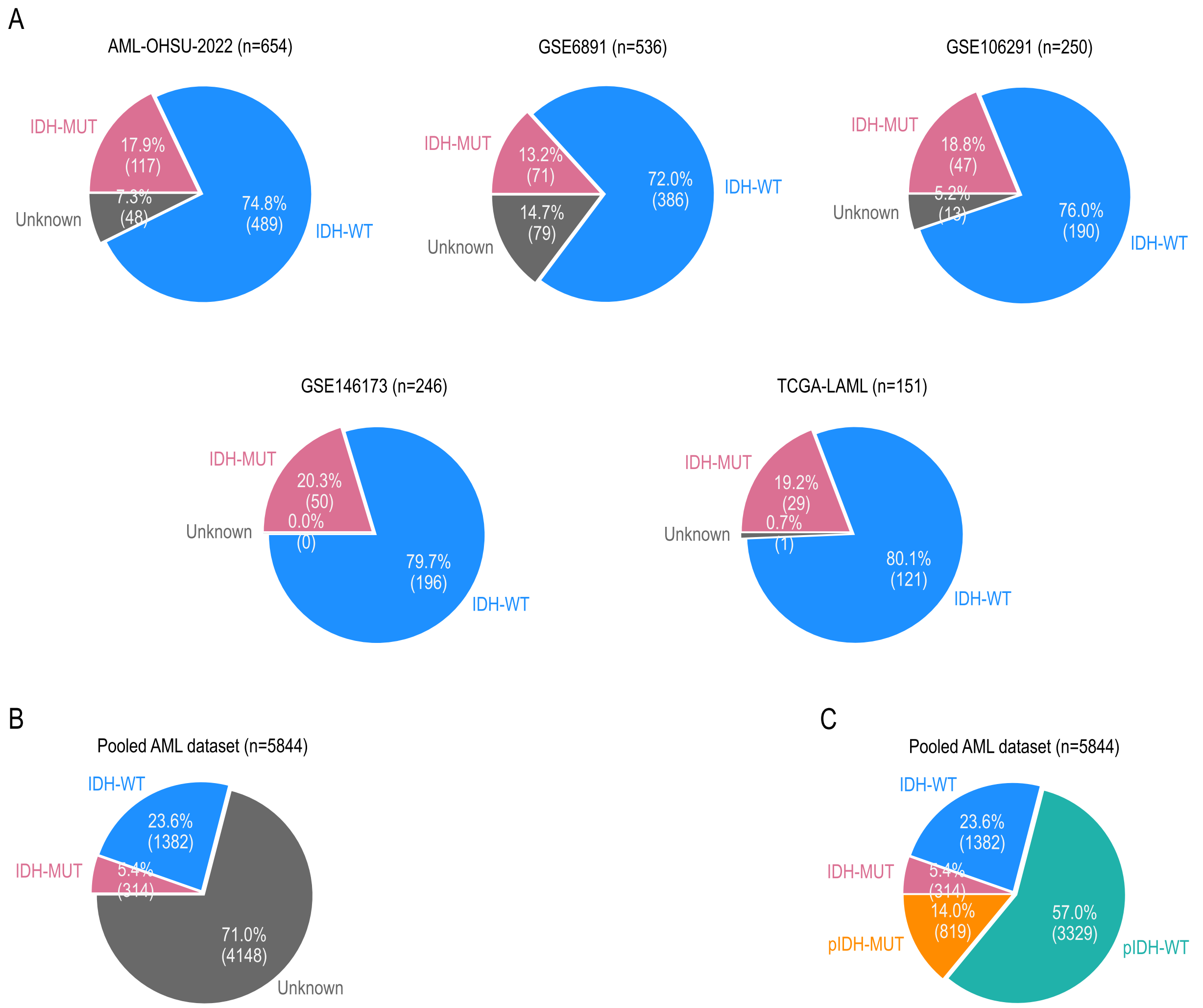

Supplementary Figure 6: Distribution of IDH status annotations and predictions across AML datasets. **A**: Pie charts showing the proportions and sample counts of IDH-wildtype (IDH-WT), IDH-mutant (IDH-MUT), and samples with unknown IDH status in each of the five original AML datasets with available mutation annotations. **B**: Pie chart summarizing the proportions and counts of IDH-WT, IDH-MUT, and samples with missing IDH status in the pooled AML compendium. **C**: Pie chart displaying proportions and sample sizes for four groups in the AML pooled dataset: confirmed IDH-WT and IDH-MUT samples (with known annotations) and predicted labels (pIDH-WT and pIDH-MUT) assigned by the logistic regression (LR) model to previously unannotated samples.

References

[1] Maxwell W Libbrecht and William Stafford Noble. Machine learning applications in genetics and genomics. *Nat. Rev. Genet.*, 16(6):321–332, June 2015.

[2] Christof Angermueller, Tanel Pärnamaa, Leopold Parts, and Oliver Stegle. Deep learning for computational biology. *Mol. Syst. Biol.*, 12(7):878, July 2016.

[3] Hui Yu, David C Samuels, Ying-Yong Zhao, and Yan Guo. Architectures and accuracy of artificial neural network for disease classification from omics data. *BMC Genomics*, 20(1):167, March 2019.

[4] Somayah Albaradei, Maha Thafar, Asim Alsaedi, Christophe Van Neste, Takashi Gojobori, Magbubah Essack, and Xin Gao. Machine learning and deep learning methods that use omics data for metastasis prediction. *Comput. Struct. Biotechnol. J.*, 19:5008–5018, September 2021.

[5] Pierre Baldi and Peter Sadowski. The dropout learning algorithm. *Artif. Intell.*, 210:78–122, May 2014.

[6] Nitish Srivastava, Geoffrey Hinton, Alex Krizhevsky, Ilya Sutskever, and Ruslan Salakhutdinov. Dropout: A simple way to prevent neural networks from overfitting. *Journal of Machine Learning Research*, 15(56):1929–1958, 2014.

[7] Sudhir Varma and Richard Simon. Bias in error estimation when using cross-validation for model selection. *BMC Bioinformatics*, 7(1):91, February 2006.
